# Tackling the escalating burden of care in Uganda: a qualitative exploration of the challenges experienced by family carers of patients with chronic non-communicable diseases

**DOI:** 10.1186/s12913-023-10337-6

**Published:** 2023-12-05

**Authors:** Lorna Montgomery, Cyprian Misinde, Alimah Komuhangi, Angela N. Kawooya, Peninah Agaba, Charlene M. McShane, Olinda Santin, Judith Apio, Christopher Jenkins, Florence Githinji, Mandi MacDonald, Florence Nakaggwa, Rose C. Nanyonga

**Affiliations:** 1 School of Social Sciences, Education and Social Work, Queen’s University Belfast, Belfast, UK; 2 Department of Population Studies, School of Statistics and Planning, Makerere University, Kampala, Uganda; 3 Institute of Public Health and Management, Clarke International University, Kampala, Uganda; 4 Centre for Public Health, Queen’s University Belfast, Belfast, UK; 5 School of Nursing and Midwifery, Queen’s University Belfast, Belfast, UK; 6 School of Nursing and Midwifery, Clarke International University, Kampala, Uganda; 7 Quality Assurance Office, Clarke International University, Kampala, Uganda; 8 Office of the Vice Chancellor, Clarke International University, Kampala, Uganda

**Keywords:** Challenges, Family carers, Uganda, Non-communicable Diseases, Burden of care

## Abstract

**Background:**

Family carers face challenges that could significantly affect their health and the health of those they care for. However, these challenges are not well documented in low-income settings, including Uganda. We explored the challenges of caring for someone with chronic non-communicable disease (NCD) in Uganda.

**Methods:**

We conducted a qualitative exploratory study at Hospice Africa, Uganda (an urban setting) and Hampton Health Center (a rural setting) in Uganda in February and March 2021. Family carers (n = 44) were recruited using snowball and purposive sampling techniques. Data were collected using focus group discussions and in-depth interviews, gathering family carer perspectives of (a) their caring role (b) their support needs, and (c) attitudes of the wider community. In total, four focus group discussions and 10 individual interviews were completed.

**Results:**

The average age of carers was 46 years old. The majority of family care was provided by female relatives, who also experienced intersectional disadvantages relating to economic opportunities and employment. Family carers carried a huge burden of care, experiencing significant challenges that affected their physical health, and material and emotional well-being. These challenges also affected the quality of care of the patients for whom they cared. Carers struggled to provide for the basic needs of the patient including the provision of medication and transport to health facilities. Carers received no formal training and limited support to carry out the caring role. They reported that they had little understanding of the patient’s illness, or how best to provide care.

**Conclusions:**

As NCDs continue to rise globally, the role of family caregivers is becoming more prominent. The need to support carers is an urgent concern. Family carer needs should be prioritised in policy and resource allocation. The need for a carer’s toolkit of resources, and the enhancement of community support, have been identified.

**Supplementary Information:**

The online version contains supplementary material available at 10.1186/s12913-023-10337-6.

## Introduction

The quality of life of people living with non-communicable diseases (NCDs) may be impacted to a large extent by the care provided by family caregivers [1]. The World Health Organization [2], reporting on long-term care needs in sub-Saharan Africa, notes that families currently provide most long-term care, and generally do so without any training or support. This practice places an unnecessary burden on caregivers, who are mostly female, perpetuating household poverty, and hindering efforts to expand education, employment, and economic opportunities. In the context of overwhelming care needs and limited healthcare resources, family carers have become pivotal in the provision of care, without which many patients would be abandoned.

In Uganda, the underdeveloped and underfunded health system, and culturally driven familial obligations, have placed a significant burden on family carers to care for the needs of chronically sick family members. Family carers in Uganda are at increased risk of developing anxiety and depression [3]. Studies have identified the extent of the burden of care, and the risk to the carer’s well-being with concerns noted in relation to social isolation and burnout [2–4]. Inadequate linkages between informal and formal care systems with multiple stakeholders and poor communication have contributed to this burden of care [5, 6]. The importance of strengthening family and community capacity to provide care [7], whilst also increasing support for carers [8], has been highlighted.

In Uganda, the burden of care for people suffering from NCDs is on the rise [9], with 27% of deaths per year attributed to chronic NCDs including cardiovascular diseases, diabetes, and cancer [10]. Typically, patients are diagnosed with an NCD with advanced disease, receiving acute care within regional or local healthcare facilities, whilst mainly receiving longer-term care at home. As the majority (approximately 84%) of Ugandans live in rural areas, most family carers are based in rural communities, which are likely to be remote and lack health service provision [11]. In this context, carers are central to maintaining the care needs of sick or disabled relatives [12, 13], with similar findings identified in other low and middle-income countries (LMIC) [14].

In recent years, Uganda has developed a robust policy framework to improve the quality of care provided to all its citizens and especially those citizens suffering from NCDs [15]. International development frameworks such as Sustainable Development Goals (SDG), especially those concerning health, have been adopted and localized within Uganda’s National Development frameworks [15]. Uganda’s vision for 2040 is to have a *Transformed Ugandan Society from a peasant to a modern and prosperous country within 30 years* [15]. Prevention and control of NCDs were noted as priority areas in the ministerial policy. Arguably, therefore, the national vision, strategic development goals, and programs provide a robust framework to improve the quality of life of the population affected by NCDs. However, despite these welcome strategic developments, the challenges faced by family carers are yet to be included in policy. This is in part due to the paucity of research into the needs of carers in Sub-Saharan Africa, with the WHO acknowledging that almost no information is available on unpaid carer needs in many Sub-Saharan countries including Uganda [2]. As such, family carer needs remain an invisible and neglected determinant of health care which deserve to be given due attention [12].

This paper reports the findings of a project undertaken in 2021, which sought to increase knowledge and understanding of the challenges family carers for patients with NCDs face in Uganda. In so doing, the voice of the family carer is presented. As NCDs continue to rise and the role of the caregiver becomes more prominent, the findings will add to the body of literature used to inform developments in policy and practice in Uganda and may have relevance to other Sub-Saharan and LMIC.

## Methods

This research was conducted as a collaborative project between (the authors’ respective universities in Uganda and UK, removed for review). A qualitative exploratory study design using both focus groups discussions (FGD) and semi-structured interviews was utilised to explore family carer experiences in two contrasting settings in Uganda. All methods were performed in accordance with the relevant guidelines and regulations of the Declaration of Helsinki with twenty thousand Ugandan shillings (about £4.50 GBP) offered to all participants as an appreciation of their time.

### Setting

Data were gathered in two purposively selected research sites: Hospice Africa, Uganda, based in the capital city of Kampala, and Share Uganda, Hampton Health Centre, based in the rural setting of Kyotera. Hospice Africa, Uganda provides palliative care to patients and training in palliative care for service providers across Africa. Share Uganda is a not-for-profit, community-based healthcare organization providing quality and sustainable health services, supporting the education of local healthcare professionals, and developing collaborative solutions to local healthcare challenges.

### Recruitment

The project was advertised through community announcements and posters, and disseminated through established health facilities within each setting. In Kyotera, these included health centres in adjoining villages, and the government central regional referral hospital. In the Hospice, project information was disseminated by health care workers. Any interested carer was asked to contact a Clinical Officer in each setting, who acted as gatekeeper and a first point of contact for carers interested in the study. Some additional participants were invited directly by the Clinical Officer through snowball sampling. Clinical Officers provided information about the project to interested carers, as appropriate and were available to answer questions. Carers elected either to participate in a FGD or to be interviewed. Carers were invited to participate if they met the following criteria:

- Aged 18 or over.
- Currently providing informal care for a patient with a diagnosed NCD receiving treatment at one of the two recruitment sites.
- Had provided care for at least one month.
- Able to communicate effectively in Luganda or English.
- Able and willing to give written or verbal informed consent.

### Data gathering

The Clinical Officer liaised with two experienced Ugandan Research Assistants (RA: AK and PA) and agreed a date and time for each FGD, which was shared with participants. The RAs travelled to the interview site and stayed for several days, meeting participants immediately prior to the FGD. The RAs were bilingual and provided participants with a Participant Information Sheet (PIS), and consent form, reading these to participants where necessary. These were made available both in English and Luganda (Luganda being the commonly spoken local language in both sites). Following the FGD, the RAs made contact with carers who had agreed to be interviewed. As before, the PIS was read to participants, and written consent was obtained prior to the interview.

Data were collected using audio-recorded FGDs and semi-structured interviews. FGDs were facilitated by two RAs. Data from Kyotera, Hampton Health Centre (Hampton HC) were collected in February 2021, and data from the Hospice in March 2021. FGDs were conducted in English, as this was the preference of all participants. RAs managed the pace and tone of the FGD to encourage participation from all carers.

In order to support carers to discuss sensitive issues and foster a balance in power relations, four FGDs were conducted in total, two in each site. Focus groups were organised by gender (i.e., one male FGD in each site (n = 7 in Hampton HC and n = 8 in Hospice) and one female group (n = 11 in Hampton HC and n = 8 in Hospice). Each FGD was facilitated in a private room within each health facility in order to minimize interruptions and ensure confidentiality. FGDs lasted between one and two hours and gathered carer perspectives of (a) their caring role (b) their support needs and (c) attitudes of the wider community. Prior to undertaking the study, the research team had completed a systematic review of relevant literature (Ref removed for peer review), with interview questions based on key themes identified in the literature.

In order to promote triangulation, in each setting, FGDs were followed by five in-depth semi-structured interviews, with carers who wished to take part in the study but not participate in a FGD. Interviews were conducted at the carers’ home and lasted one to two hours. A similar interview schedule to that used in the FGD was used to further explore carer challenges. (COVID-19 prevention measures were followed throughout all data collection processes). The interview schedules for both the FGD and individual interviews were developed for this study and can be found in the supplementary material.

### Data analysis

Research data were transcribed verbatim by two RAs. Anonymised, cleaned transcripts were uploaded to a shared online platform which was accessed by all team members; transcripts were checked for discrepancies and collaboratively analyzed using thematic analysis. In this process, the wider study team, comprising an interdisciplinary group of academics from both UK and Uganda, were organised into four subgroups, each consisting of at least one Ugandan and one UK based team member with each group taking a proportion of the data to analyse, up to the point of identifying sub-themes. Through online meetings, thematic analysis was completed collectively within each group. Braun and Clarke’s [16] six-step approach was followed. Each subgroup familiarized themselves with the whole data set before focusing on their own subset of data; next, initial semantic data-driven codes were generated for each subgroup; following this, codes were then collated into broad patterns or themes for each subgroup; these themes were then reviewed and major themes and subthemes were identified. Each subgroup then presented a list of their themes and subthemes associated with their data set to the wider team, and these were collated, themes and subthemes were identified across the whole data set through iterative facilitated discussions. Finally, the analysis was agreed and written up. This procedure combined the interviews and FGD within the analysis, enabling the team to make sense of collective or shared meanings and experiences of carers across all data sets.

### Research rigor

The following steps were utilised in promoting rigor and trustworthiness. All data collection processes implemented a standardised approach using a topic guide. Data acquisition methods were triangulated (FGD and individual interviews). The RAs were Ugandan nationals, who were familiar with cultural norms. All research team members received training in research methods during a focused 3-day workshop at the outset of the project. Data analysis followed a systematic procedure. Preliminary findings were critically discussed at various points with the research team. Additionally, after data analysis was completed, the RAs travelled back to each research site to enable data checking with key stakeholders, including the Clinical Officer and research partners. Results are reported following the Consolidated Criteria for Reporting of Qualitative Studies (COREQ) guideline.

## Results

Family carers (n = 44) participated in the study across both sites (n = 34 for FGD) and (n = 10 for individual semi-structured interviews).As noted in Table 1, of the 44 carers who participated across both settings, n = 26 (59.1%) were female and n = 18 (40.9%) were male. The average age of participants was 46 years old. Prior to taking up the caregiving role, many carers worked in either informal or formal employment. This included carers who were: peasant farmers, road sweepers, teachers, businessmen, and women, shopkeepers, and mechanics. One carer was retired with his current income source unknown. Family carers in the study provided care to patients suffering from a range of conditions including sickle cell disease, cancer, epilepsy, mental health-related illnesses, Alzheimer’s disease, dementia, multi-morbidity (cancer/HIV/AIDS), liver cirrhosis, diabetes, and hypertension.

**Table 1:** Participant details

Sex	Age in years	Occupation	Relationship to patient (s)	Years of care
Female	34	Businesswoman	Daughter In-Law	2
Female	45	Businesswoman	Wife	13
Female	37	Peasant Farmer	Daughter	10
Female	60	Peasant Farmer and Tailor	Mother	5
Female	50	Peasant Farmer	Mother	3
Female	71	Peasant Farmer	Daughter	27
Female	48	Peasant Farmer	Daughter	NK
Female	46	Peasant Farmer	Sister	NK
Female	41	Peasant Farmer	Mother	NK
Female	30	Road Sweeper	Mother	3
Female	25	Shop Keeper	Mother	2
Female	34	Teacher	Landlady	15
Female	48	Teacher	Sister	2
Female	25	Unemployed	Dual Carer (Granddaughter, Daughter)	2
Female	61	Unemployed	Grandmother	2
Male	51	Computer analyst	Son	10
Male	70	Brewer	Husband	5
Male	27	Businessman	Son	*NK
Male	51	Businessman	Son	13
Male	71	Businessman	Dual Carer (Husband, Father)	43
Male	32	Peasant Farmer	Dual Carer (Grandson, Father)	5
Male	73	Peasant Farmer	Son	15
Male	45	Peasant Farmer	Father	NK
Male	34	Peasant Farmer	Son	4
Male	36	Peasant Farmer/Retired Teacher	Father	3

The duration of care ranged from two months to 43 years. Participants engaged in multiple caregiving roles with tasks including: provision of basic care needs: food provision; maintaining patient hygiene and general wellbeing; administering medication and managing symptoms including measuring blood pressure, and monitoring patients movement. Carers provided companionship, spiritual, psychosocial and emotional support, whilst also communicating with extended family members and health and social care professionals. These activities were largely similar across age groups, male and female carers, as well as in both rural and urban settings.

### Family carer experiences

To a large extent, there was continuity in the key themes identified across settings. Participants portrayed their caregiving role as both fulfilling and challenging, however, in both settings, comments on the challenges of caregiving far outweighed those comments relating to positive aspects of caregiving. This paper presents participant challenges according to the following themes (1) emotional challenges (2) challenges relating to carers’ health and well-being (3) financial and employment challenges (4) logistical and access challenges (5) marital and social disruption (6) health literacy challenges, and (7) the role of the community.

The discussion of each theme will be supported by indicative quotations, presented with the following anonymous identifiers which included the relevant health facility’s name.

**Table:** 

KEY	Participant categories
**MIDI**	Male Individual Interview
**FIDI**	Female Individual Interview
**MFGD**	Male Focus Group Discussion
**FFGD**	Female Focus Group Discussion

### Emotional challenges

Whilst some participants perceived their caring role to be fulfilling, the majority of carers presented caregiving as a difficult experience that induced feelings of sadness, anxiety, worry, frustration, and guilt. Feelings were often attributed to a lack of support, concerns around finances, limited knowledge of the illness and its progression, and complications of the disease which often engendered feelings of helplessness. Emotional challenges were also associated with strong feelings of empathy for the patient, and with providing end-of-life care for some patients. Conflict arising from stresses within the caring relationship were also identified. Care was generally provided either in the patient’s or caregiver’s home, with carers also worrying about their ability to provide financially and practically for members of their own household as well as caring for the patient.

What can worry you most is that maybe my children have not eaten, or my children don’t have sugar, you can be worried that now I am here, but my children are lacking what to use at home, things like that. (FIDI Hospice 1)

Participants described feeling ‘great sadness’ at the patient’s pain and suffering. Often conveying a strong sense of empathy for the patient.

I felt pain as a parent, I had hope that maybe he would have some medicine to at least help him reduce the size of the tumor, but he told me that there was nothing. (FIDI Hospice 2)

*These days every time I bathe her, sadness comes in when she cries, then I also get sad*. (FIDI Hospice 1)

### Challenges relating to carers’ health and well-being

Participants reported that caring often had a negative impact on their own health and well-being. Carers reported physical ill-health, which negatively impacted their caregiving capacity. These negative health outcomes further affected the quality of care patient’s received.

Yes, I have chest pain and I have ulcers…. That’s the challenge I find, carrying an adult has caused me chest pain. …Ulcers are due to worrying about my sister. (FIDI Hospice 1)

Participants also expressed fatigue, experiencing inadequate sleep due to the twenty-four-hour nature of their caring responsibilities.

Healthwise challenge is sleepless nights because you have to be there and be attentive at all times, you can’t get that rest that is needed. (FIDI Hospice 5)

*A lot of tiredness since the carers are alone and get tired.* (FFGD Hampton HC 6)

Caring did not stop even for those who were pregnant, with one participant expressing concern about caregiving responsibilities whilst also being pregnant.

Like last time, the time I took care of X, I was pregnant for this child … generally, it was so hard for me, and I was also worried of the child inside me to keep it alive. (FIDI Hospice 3)

Participants also reported concealing their health needs from both the patient and from health providers in order to continue their caring role, afraid that disclosing personal sickness might result in the removal of the patient.

Now, today the doctor came but I feared to tell him about my sickness. (FIDI Hospice 1)

### Financial and employment challenges

Pervasive financial challenges and constraints were identified across both settings. The chronic and often long-term treatment required for many NCD patients contributed to extensive costs, which were assumed by carers. Carers reported increasing financial difficulties with ‘dwindling resources’ as their caring responsibilities continued over time. Carers often struggled to provide for a patient’s basic needs including the need for food, medicine, transport, and medical treatment.

Because of lack of money, I cannot afford to buy what she needs. (FIDI Hospice 4)

As noted, some carers had to support two households: the carer’s family home where their children or other family members often remained, and the patient’s home,

I earn little, and I have to make sure the money I earn is enough to care for my mother and my family. (MIDI Hampton HC 4)

Moreover, financial difficulties were exacerbated by carers’ limited time for income-generating activities and paid employment. Carers also reported that they could no longer save money which affected their potential to invest and conduct future business.

The changes are brought about by the patient’s illness. … l cannot even save any money. Everything I earn goes to caring for the patient and the family. (MIDI Hampton HC 4)

Some participants stopped working entirely, others indicated that they lost their jobs and or businesses, while some described trying to maintain employment whilst acting as primary carer, but eventually having to leave work.

I am a farmer and it’s raining, and people are planting but I have given up everything for the sake of my sister. (FIDI Hospice 1)

Carers who maintained jobs struggled with care coordination in the absence of other much-needed support and, as noted, often eventually gave up their employment.

I got a challenge when l used to work with X (Organization) and had a very busy work schedule by then. During that time when l would be away working, my son would get attacks every two weeks and would be taken for blood transfusion. This forced me to resign from my job and come back home to take care of my son. My coming back home helped me a lot because if l had not done so, my son would have been dead a long time ago. (MFGD Hampton HC 3)

I used to work and make money, but now l have to focus on the patient and myself. And l used to save some money as well, but now everything goes into caring for the patient and myself. … (FIDI Hampton HC 2).

Participant strategies to address financial constraints included borrowing money and securing medication through credit. Carers also sought financial support from family or community members, resulting, on occasion, in alienation from their social networks.

We cannot work to take care of ourselves… that causes over-dependency and always asking for help for two people (FFGD Hampton HC 7).

People are scared of us, and they think we are going to beg them. (FFGD Hampton HC 6)

### Logistical and access challenges

The impact of logistical challenges was identified across both settings. Many carers discussed the challenges related to transport, balancing work and carer roles, access to health facilities and supplies as well as high costs associated with multiple appointments and travelling long distances. The majority of participants described significant challenges in relation to transport, particularly transporting a patient to hospital, with associated fuel costs. Travel costs were exacerbated by multiple appointments and the need to travel long distances.

The challenge is transport, the patient is for carrying so she needs a means of transport from home to the hospitals. That’s the challenge we face, then carrying because you have to carry her to the car, that’s the major problem when going to hospitals. (FIDI Hospice 1)

*Transport from one facility to another is also another problem in cases of referrals. Sometimes he (the patient) becomes very sick in the middle of the night and you have to find a way of reaching the hospital. He cannot walk really well anymore, he is weak as a man, so many things a man has to do, I have to do instead.* (FIDI Hampton HC 1)

Participants also reported experiences reflecting inequalities in access to healthcare, and medical supplies, for example having to secure supplies outside the public healthcare system.

If l could get assistance in terms of accessing care in those powerful health facilities so that the patient can be assessed and given medication that may help relieve her condition other than just seeking care from the local health care facilities in this community.(MIDI Hampton HC 2).

Decisions about which health facility to access were also determined by finances.

Money determines where to take the patient,” (MFGD Hampton HC 3).

*You look into your earning and see what money you can manage; it can be that the facility near you is expensive for you to manage. There is where you can get free support and so you go there.* (FFGD Hospice 1)

### Marital and social disruptions

Participants described significant disruptions to their family life including impact on their marital relationships, which resulted from the emotional, sexual, and social disruption of the marriage. This was often attributed to a wife caring for another family member and being seen to neglect her husband or family. This was perhaps most obvious when carers were required to relocate to the home of the patient.

I gave up all that and said ‘let me go and look after my sister’. So that’s a problem because I no longer have anything, I left my home. (FIDI Hospice 1)

*If it is your family, you give it less time because you must be with the patient you can’t mix the two. …and basically the patient needs more time than the others.* (FIDI Hospice 5)

In some instances, marital disruptions ensued when female carers were blamed for the child’s illness as if they were the direct cause of it. One participant reported:

The challenges I get is that of home misunderstandings because some men think that when an illness comes, just like that of my child, then that child is not his. They think that maybe the child’s illness was inherited from the child’s family because to men, the sick children are not theirs, be it sickle cells or what, they (men) are not responsible.’ (FIDI Hospice 2).

### Challenges relating to health literacy

A lack of prior training or skill in providing care was identified with carers often taking on their caring role in an unplanned way. Carers identified challenges associated with limited knowledge of the illness and felt ill-equipped to deal with issues such as a nosebleed for a patient with sickle cell disease or back pain. They identified the need for information about the disease, disease progression, and patient management.

However, some carers described positive experiences in developing their caregiving skills and increasing their knowledge of the treatment pathways, for example, one carer described how she learnt to manage the patient’s diet and medication.

Because they tell me how to administer the medicine in time, to measure for her what to drink in time and the small meal she can manage to eat. Now I am used to that at night.

…. I understand all that very well. (FIDI Hospice 1)

Whilst not explored directly, the ways in which carer’s conceptualised illness became apparent. Some carers accepted the medical model, others concurred with a more traditional cultural belief system. These beliefs influenced the patient’s journey including treatment choice and adherence to treatment regimes.

For many years, we have been giving her herbal medicine. But because herbal medicine was very expensive, we resorted to taking her to x (health centre). (MIDI Hampton HC 1)

### The role of the community

Many participants acknowledged the role that the community, including neighbours, friends, or religious groups, played in supporting them and their patients. However, the level and nature of support varied. Support by the church was appreciated, but some religious leaders were perceived as being exploitative; for example, emphasizing the need to donate money to the church at the expense of providing spiritual care. Community support included encouragement,

The community feel pity on me…. Some community members encourage us. (MFGD Hampton HC 3)

Help with financial and practical needs was also identified.

I get help from my neighbor in (the) form of communication, transport and connections for blood for transfusion. I get help in terms of money for treatment, food and other things from my daughters. (MFGD Hampton HC 3)

Since my patients are fond of disappearing, the community helps me in finding them and bringing them back to me. Sometimes the epileptic patient disappears, and l look for her in vain, then eventually, you see a motorcyclist bringing her back at 9pm. (FGD Hampton HC 8.)

However, some carers reported being discouraged as a result of limited help or negative community attitudes:

The only community member who has been of great help is Mr. X and his wife: He has helped us in various ways; from bearing with us when school fees are due, providing transport to the facility where l cannot afford and money for medication as well. (FIDI Hampton HC 1)

## Discussion

This paper reports on the challenges of caring for someone with a chronic NCD in Uganda from the perspective of family carers. Utilising FGDs, and individual interviews, family carers (n = 44) from rural and urban settings, shared their experiences and challenges of providing care for a family member. Carers described emotional challenges, challenges relating to their physical health and wellbeing, financial and employment challenges, logistical and access challenges, impact on marital and social relationships, poor health literacy and the role of the community. These findings address a gap in the literature with previous research on care for people with NCDs in Uganda focused on formal health care systems performance [9], with limited attention paid to non-healthcare determinants such as informal care [1, 2].

In exploring the perceptions of carers, it is evident that family carers carry a significant burden in caring for patients suffering from NCDs. As NCDs continue to rise, the role of family caregivers is becoming more prominent, however, the WHO argue that family-based care, in its present configuration, is unable to deliver good-quality and integrated long-term care. The WHO recommends that carers’ needs should be prioritised in order to promote the development of sustainable and equitable long-term care systems and SDGs [2]. The findings in this study identify the nature of the caregiving burden in seven core areas, and can inform the development of carers’ policy and service provision.

As documented in other studies of family carers in Uganda [17–21], carers identify that caregiving engenders a strong emotional response characterised by anxiety, worry, frustration, and guilt. In this study, these emotional responses were attributed, in part, to a combination of empathy felt for the patient alongside carers’ inability to provide quality care due to limitations in support and service provision.

Carers also reported a negative impact on their own physical health and wellbeing, with limited time to prioritise their own self-care needs or engage in self-care practices, resulting in fatigue and health complications. As identified in other studies, carers require support to balance their caregiving responsibilities whilst promoting their own health and wellbeing [20].

Challenges associated with inadequate finances were identified by the majority of carers. Concurring with other reported caregiver experiences in Uganda [20] and in other LMIC countries [22], factors contributing to financial difficulties included loss of income-generating opportunities, especially for carers who had left jobs to take up full-time caring roles, and dual expenditure for carers with financial responsibility for both their own and the patient’s household. This financial burden included direct costs associated with providing care and opportunity costs for missed education, employment and economic engagement. In this study, the lack of adequate finances led some carers to adopt risky and unsustainable coping strategies such as borrowing money, and ‘begging’, with a consequent risk of alienation from social networks. It has been noted that in Uganda and throughout sub-Saharan Africa, the majority of family care is provided by female relatives, who also experience intersectional disadvantages relating to economic opportunities and employment [2].

Related to the financial concerns of carers, logistical problems, including transporting patients to mandated health facilities, was highlighted as a major challenge. Similar carer challenges have been reported in other studies in Uganda [17–19]; throughout sub-Saharan Africa [2], and in wider LMIC settings [14]. Oxfam reports that poor quality health facilities along with costly services converge to discourage patients from seeking professional care, and point to the need to invest in care-supporting services and infrastructure [23].

Consistent with other Ugandan studies [19, 24], carers also reported marriage and social disruptions, often prioritising the needs of patients over their own families, with resultant strain in relationships. An added dynamic, also noted in studies of mentally ill patients in Uganda [25], was the husband’s insistence that he was not the biological father of the sick child, placing blame for a hereditary condition elsewhere. Moreover, as identified elsewhere [26], dwindling support networks were evident as carers experienced isolation from relatives and friends as caregiving relegated them to a status of dependency.

Contributing to carer challenges was the issue of poor health literacy; many participants became carers without training, experience, or knowledge of what to expect. Most carers identified the need for information about the disease and its progression, and patient management. Improving carers’ competency and motivation can enhance health outcomes for patients (and themselves) and promote health system sustainability [27–29].

Finally, the experience of family carers need to be understood within the wider community context. Community responses appeared to vary, with some carers identifying both practical and emotional support, whilst others identifying limits to the support offered. One reason given for this was community member concerns that carers would make financial demands of them. The role of churches was highlighted here, as elsewhere [25], with spiritual support deemed to be variable.

### Study limitations

This qualitative study gathered the perceptions of family members caring for someone with an NCD. As a qualitative study with a purposive sampling method and inclusion of 44 carers, the results are limited to selected participants in two distinct locations. However, in presenting the voice of the carer, this study has conveyed first-hand the needs, experiences, and complexities of caring for a family member with NCD.

### Conclusion and recommendations

This study presented the self-reported concerns of family carers in Uganda. In fulfilling their caring role, family carers experienced emotional difficulties, challenges relating to their physical health and wellbeing, financial, employment and logistical challenges. Carers were concerned about limits to their health literacy, they described caring as having a negative impact on their marital and social relationships, and identified a mixed community response to both the patient and themselves as carers.

Within the context of these care-giving challenges, this study demonstrated that patients’ treatment and care was fundamentally influenced by their home and family circumstances, perhaps more so than their engagement with formal health systems. However, carer needs were not being addressed. Whilst the Ugandan government is making progressive policies aimed at the prevention, and treatment of NCDs in formal health systems [15], the role and the needs of family carers have not been well understood and have not yet been integrated into relevant policies [3, 30]. As a result, limited support, resources, and social protection is provided to family carers for patients with NCD [31]. Knowledge generated in this study should be used as evidence to begin integrating family carer needs into mainstream health and social care policies, with the following recommendations identifying mechanisms to address carer needs.

Consideration should be given to supporting carers financially, whilst providing practical assistance with the daily tasks of caring, central of which is transportation to health facilities. Systems should be in place to ensure that family caregivers in Sub-Sahara Africa have access to the resources, information, and training to fulfill their role [2]. Carers’ health literacy would be enhanced by providing information about the illness, treatment pathways, and practical issues. Building on similar initiatives [32], a toolkit of resources providing information and psychosocial support to informal carers should be developed as a priority.

Moreover, health-sector training appears biased towards the medical model in Uganda and other LMIC [33] and does not necessarily view patients’ within their family or home context. Thus, training for healthcare providers should provide a more holistic, systematic view of patient’s treatment and care whilst recognizing carer’s role and support needs.

In the context of overwhelming care needs and limited resources, there is a need to strengthen community support [2, 7]. Community-strengthening processes should be considered, as documented and implemented in HIV care [6]. The potential for community-systems to act as an entity, distinct and separate from family carers, which could contribute to the patient’s needs, should be considered [7].

Finally, further research should include studies that trial interventions on supporting carers in both urban and rural contexts. This research should be prioritized within future funding streams, adding to the development of evidence-based programs to support carers.

### Electronic supplementary material

Below is the link to the electronic supplementary material.

Supplementary Material 1

## Data Availability

The datasets used and/or analysed during the current study are available from the corresponding author on reasonable request.

## Abbreviations

**NCDs**: Non-Communicable Diseases
**LMIC**: Low and Middle-Income Countries
**NPA**: National Planning Authority
**FGD**: Focus Group Discussions
**RAs**: Research Assistants
**COREQ**: Consolidated Criteria for Reporting of Qualitative Studies
**MIDI**: Male Individual Interview
**FIDI**: Female Individual Interview
**MFGD**: Male Focus Group Discussion
**FFGD**: Female Focus Group Discussion
**DfE**: Department for the Economy
**CGRF**: Global Challenges Research Fund
**GAC**: Global AIDS Coordinator
**PEPFAR**: President’s Emergency Plan for AIDS Relief
