# Supplementary material for: Tackling the escalating burden of care in Uganda: a qualitative exploration of the challenges experienced by family carers of patients with chronic non-communicable diseases
Source: BMC Health Serv Res. 2023 Dec 5;23:1356. doi: 10.1186/s12913-023-10337-6 (PMC10696811; doi:10.1186/s12913-023-10337-6)
Supplement: Supplementary file 1 — Supplementary Material 1 [file 12913_2023_10337_MOESM1_ESM.docx]

**Focus Group Schedule for Informal carers: English version**

This tool is to be used for focus group discussion with informal carers in Uganda. These participants in the FGD are expected to provide general information about the type of people’s experience with care, challenges carers face, types of support needed by carers.

**Instructions to Interviewer**

• Introduce yourself and the purpose

• Explain the objective of the study and key issues of discussion

• Invite the participants to introduce themselves, providing basic demographic information: age, occupation, place of residence and marital status?

• Take note of the details of category of participants who are in the FGDs.

1) Can everyone tell me a few things about their caregiving experience?

*Prompt; who do you care for, what type of illness does the patient have, how long have you been caring for the patient

2) How are carers decided/chosen in your community? What factors are considered before one becomes a care? Can each of you tell me how you become the main person to provide care for the patient?

*Prompt: How was it decided that you would give support?/ decisions based on gender/ age/ birth rank in a family, marital status, occupational status, voluntary decision, personal decision, economic ability, etc

3) What type of care do people provide to the patients with NCDs? Probe for physical /food/ counselling, financial/ etc

*Prompt: Physical/ food/ nutrition / counselling / washing / financial/ companionship/

4) What other roles do other members of the family/relatives/ community play in supporting the carer? Probe for the type of support from other family members, relatives, friends, community

*Prompt: Do other family members help, do other community members help/ is there any financial support

5) What are the likely impact of caring on the carer? Probe for health, financial, occupational/ spiritual/ relationships impacts

*Prompt: Physical/ mental health/ depression/ sorrow/ social,/finance / employment/education

6) Can you tell me the greatest issues or problems that people face in caring for a family member?

*Prompt: What are the main issues you worry about? What are the main issues you need help to manage? Health care fees/ finance/ breadwinner cannot work/ limited access to health care/geographical issues/education.

7) Where do patients of NCDs seek help for their illness in your community? What are the factors considered when choosing a place of care in your community?

*Prompt: Traditional medicine/ medical doctors/faith healing

8) What does the community think about carers in your community?

Prompt* Probe for praises/ provide sympathy/ experience of prejudice/stigma/support.

What do people in your community say about carers?

Prompt* Probe for praises/ provide sympathy/ experience of prejudice/stigma/support.

9) What support do the carers need to effectively provide care to patients in your community? What support would help you in your caring role?

*Prompt: Emotional/ practical/financial/spiritual

10). Tell me about the organisations that provide care to patients with NCDs in your community? What kind of support do they provide?

*Prompt: financial/ emotional/ practical/ help with relationship difficulties

11) Are there any other issues which you would like to discuss which is relevant to this topic?

**Schedule for Informal carers individual interview: English version**

Individual interview with family carer:

Expanding on some of the things that was said in the focus group.

This tool is to be used to interview key informants who are informal carers. These respondents are expected to provide information about their experiences in caring, the type of support they need, challenges they face, changes they have experienced while caring for the patient and what they think should be done to support informal carers.

Instructions to Interviewer

• Introduce yourself and the purpose

• Explain the objective of the study and key issues of discussion detailing key issues of consent and confidentiality. Invite the participants to introduce herself or himself, providing basic demographic information: age, occupation, place of residence and marital status? Take note of the details of category of respondent your are interviewing

1) Tell me about your caring experiences?

*Prompt; who do you care for, what type of illness does the patient have, how long have you been caring for the patient

2) Can you tell me how you become the main person to provide care for your ….. (you can use title of the person provided (daddy, mummy, uncle, son etc? what made you to decide to take care of this patient?

*Prompt: How was it decided that you would give support?/ decisions based on gender/ age/ birth rank in a family, marital status, occupational status, voluntary decision, personal decision, economic ability, etc

3)a What type of care do you and your family provide to the patient?

*Prompt: Physical/ food/ nutrition / counselling / washing / financial/ companionship/

3b. In a typical day of 24 hours, describe for me the types of care you provide to your …..( daddy, mummy, uncle, son etc)

4) What help do you get in caring from your family/ community members? How frequently do you get the support?

*Prompt: Do other family members help, do other community members help/ is there any financial support

5) Ever since you started caring for your….( daddy, mummy, uncle, son etc) what changes have you experienced as a person?

*Prompt: Physical- / mental health/ depression/ sorrow/ social,/finance / employment/education. (Have you lost or gained weight? have your sleeping patterns changed, do you fall sic often etc)

5b) what do you think has caused the changes in your life you have mentioned?

6) Can you tell me the greatest issues or problems you face while providing care for your …..….( daddy, mummy, uncle, son etc) ?

*Prompt: What are the main issues you worry about? What are the main issues you need help to manage? Health care fees/ finance/ breadwinner cannot work/ limited access to health care/geographical issues/education.

7) Where has your ……( daddy, mummy, uncle, son etc) the patient sought help for his or her illness? What factors does he considered while choosing the place of care you have mentioned?

*Prompt: Traditional medicine/ medical doctors/faith healing.

7b) What are the challenges if any faced in supporting your ….to access those places of care? How did you overcome them?

8) What do people in your community say about you as a carer? Probe for etc) How has the community thought of you or treated you as a carer?” How has the community thought of or treated the patient?

*Prompt: Have you experienced prejudice/stigma/ support/ praises/ provide sympathy/? Has the patient experienced prejudice/stigma/ support/ praises/ provide sympathy?

9) What support would help you in your caring role?

*Prompt: Emotional/ practical/financial/spiritual

10). As a caregiver have you received any support from Uganda hospice or any other health facility? If yes which kind of support?

*Prompt: financial/ emotional/ practical/ help with relationship difficulties

11) Are there any other issues which you would like to discuss which is relevant to this topic?
